# Exploration of collagen cavitation based on peptide electrolysis

**DOI:** 10.1038/s41598-021-96533-y

**Published:** 2021-08-24

**Authors:** Rui Zhai, Hui Chen, Zhihua Shan

**Affiliations:** The Key Laboratory of Leather Chemistry and Engineering of Ministry of Education, Sichuan University, Chengdu, 610065 China

**Keywords:** Peptides, Proteolysis

## Abstract

Electrochemical modification of animal skin is a new material preparation method and new direction of research exploration. In this study, under the action of the electric field using NaCl as the supporting electrolyte, the effect of electrolysis on Glycyl-glycine(GlyGl), gelatin(Gel) and Three-dimensional rawhide collagen(3DC) were determined. The amino group of GlyGl is quickly eliminated within the anode region by electrolysis isolated by an anion exchange membrane. Using the same method, it was found that the molecular weight of Gel and the isoelectric point of the Gel decreased, and the viscosity and transparency of the Gel solution obviously changed. The electrolytic dissolution and structural changes of 3DC were further investigated. The results of TOC and TN showed that the organic matter in 3DC was dissolved by electrolysis, and the tissue cavitation was obvious. A new approach for the preparation of collagen-based multi-pore biomaterials by electrochemical method was explored.

## Introduction

Porous materials have been extensively studied and widely used in functional materials for applications such as toxin adsorption^1^, drug release^2^, oil–water separation^3^, sound insulation^4^ and so on. In addition to natural formation, a variety of organic porous materials mainly come from artificial synthesis and rarely from chemical carving^5^ or cavitation treatment of solid materials^6^. In the process of making leather from animal skins, the hair, fat, glands, interfibrillar substance, and some non-collagenous components are removed to obtain the separation of the fibers and left enough pores in the rawhide, which provides the tanning agent enters for tanning and satisfies the soft and plump leather senses. For more than one thousand years, because the pores was mainly from the splitting of the collagen fiber structure by lime^7^, the resulting sludge pollution was difficult to deal with and limited now^8^. However, animal skins not only are abundant renewable resources but natural and safe biomaterial. Collagen is biodegradable and has good biocompatibility, which has been widely used in biomedical materials^9–11^. Exploring non-chemical treatment of rawhide collagen to expand the inner pores and preparing biologically relevant functional porosint to store water, medicine, gas, and energy, it will be superior to other gelatin-based porous materials in both physical mechanical properties and solubility resistance^12^.

There have been many studies on the function damage and strength decrease of protein under the action of oxidant, which leads to the structural change and dissolution loss of amino acids^13–15^. Electrochemistry is a science that studies the transformations between chemical energy and electric energy and the related laws in the process of transformation. Electrochemical processes can achieve direct or indirect oxidation and reduction reactions, polymerization and dissociation, the killing of biological organisms, and phase separation under room temperature and atmospheric pressure, and is applicable to a wide range of objects^16–18^. Therefore, electrochemical technology has been widely used in many fields. Under the influence of an electric field, on the one hand, the polymer is polarized by potential to produce conformational or assembly changes^19^; on the other hand, the redox effect by electron action modifies the microstructure of macromolecules^20^. Three-dimensional rawhide collagen (3DC) is a macromolecular amphoteric polymer electrolyte with natural woven structure. To understand the changes in 3DC exposured to an electric field, some experiments and analyses were performed by exploring GlyGl and gelatin electrolysis. According to the experimental results, the organizational structure of the cowhide collagen was separated and cavitated by electrochemical "engraving".

## Experiment

### Main experimental reagents and instruments

#### Main experimental materials and reagents

GlyGl, gelatin (Gel), B type, was obtained from Chengdu Kelong Chemical Company. NaCl, ninhydrin, ascorbic acid, and D_2_O were analytically pure and purchased from Chengdu Jinshan Chemical Reagent Co., Ltd.

Anion and cation exchange membranes: styrene series, transmittance ≥ 90%, film surface resistance ≤ 12 Ω·cm^2^, were obtained from Zhejiang Environmental Protection Water Treatment Co., Ltd.

Preparation of 3DC: Salted goat skin was obtained from Chengdu, China, soaked, and degreased using surfactants. The hair, epidermis and noncollagenous components in raw skin were removed with lime and enzyme according to the requirements before tanning; the 3DC raw material was then obtained after washing, adjusting the pH with 1% HCl to approximately 5.0, wringing, and shaving the skin to a thickness of 0.65 ± 0.05 mm; it was then refrigerated for later use^21^. The auxiliaries for the preparation of 3DC were industrial grade and came from Chinese leather chemical companies.

#### Main instruments and equipment

The LK98BII-type microcomputer electrochemical analysis system was from Shanghai Analytical Instrument Co. Ltd. The titanium electrode and graphite electrode were obtained from Shanghai Liyou Electric Co., Ltd.; 25 mL electrolytic cell, homemade; the DF-101SJI heat collection constant temperature heating magnetic stirrer was obtained from Zhengzhou Great Wall Science and Industry Co., Ltd.

### Electrolysis of GlyGl

#### Electrolytic experimental conditions

All electrolysis experiments used NaCl as the supporting electrolyte and indirect oxidant, and experimental methods #1 ~ #3 are presented in Table 1. In experiment #1, 200 mL of a solution with 2.5 g/L of GlyGl and 20 g/L of NaCl was added to the cell without an ionic exchange membrane.

**Table 1:** Electrolytic experiment setup of GlyGl.

1# (No membrane)	2# (Cation membrane)		3# (Anionic membrane)	
Anode + cathode	Anode	Cathode	Anode	Cathode
GlyGl	GlyGl	DIW	GlyGl	DIW

In experiments #2 and #3, 100 mL of solution with 5 g/L of GlyGl or Gel was added to the side of the electrolytic cell with the ionic exchange membrane, and 100 mL of deionized water was added to the other cell. The concentration of NaCl was 20 g/L on both sides.

**Figure 1:**
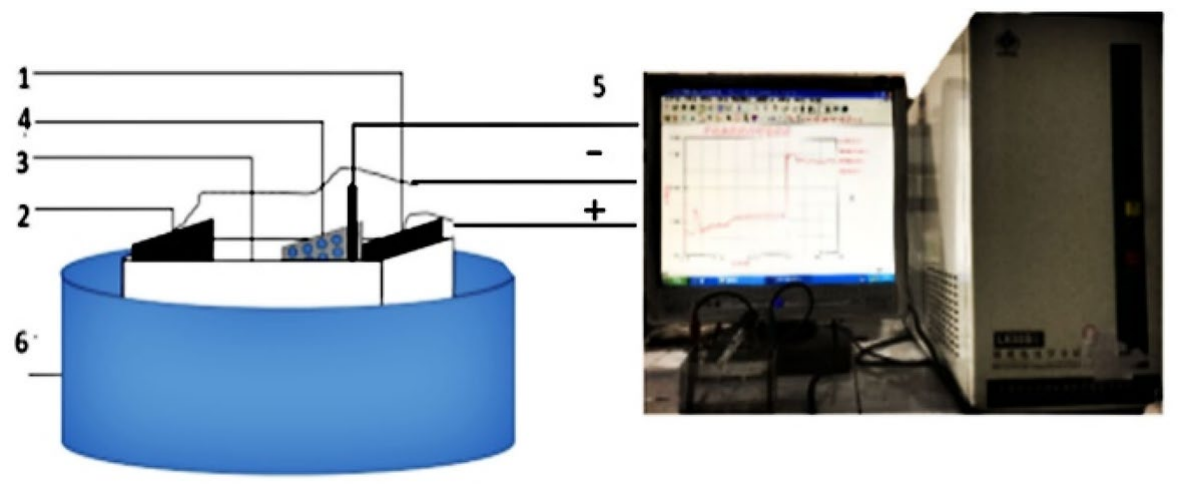
Diagram of the electrolytic reaction device. Anode plate 2-Cathode plate 3-Electrolytic cell 4-ion exchange membrane. 5-Reference electrode 6-Constant temperature water bath.

In a 250 mL electrolytic cell, a titanium plate was used as the cathode, graphene was used as the anode, and a saturated calomel electrode was connected as the reference electrode to determine the pH of the electrolyte. The reaction device is shown in Fig. 1. In this experiment, the temperature was 22 °C, and the electrolyzer was placed on a constant temperature magnetic stirrer in a water bath (DF-101SJI, Zhengzhou Great Wall Science, Industry and Trade Co., Ltd.). The current and voltage in the electrolysis process were adjusted by an LK98BII electrochemical analyzer, maintaining an average current of 15 mA·cm^−2^ and a voltage of 12 ~ 13 V. The total electrolysis time was 3 h. After each sampling, the electrolyte was removed for analysis at the completion of an electrolytic experiment, and the same experiment was repeated by adding new liquid for a parallel experiment.

#### Electrolyte pH control

**Figure 2:**
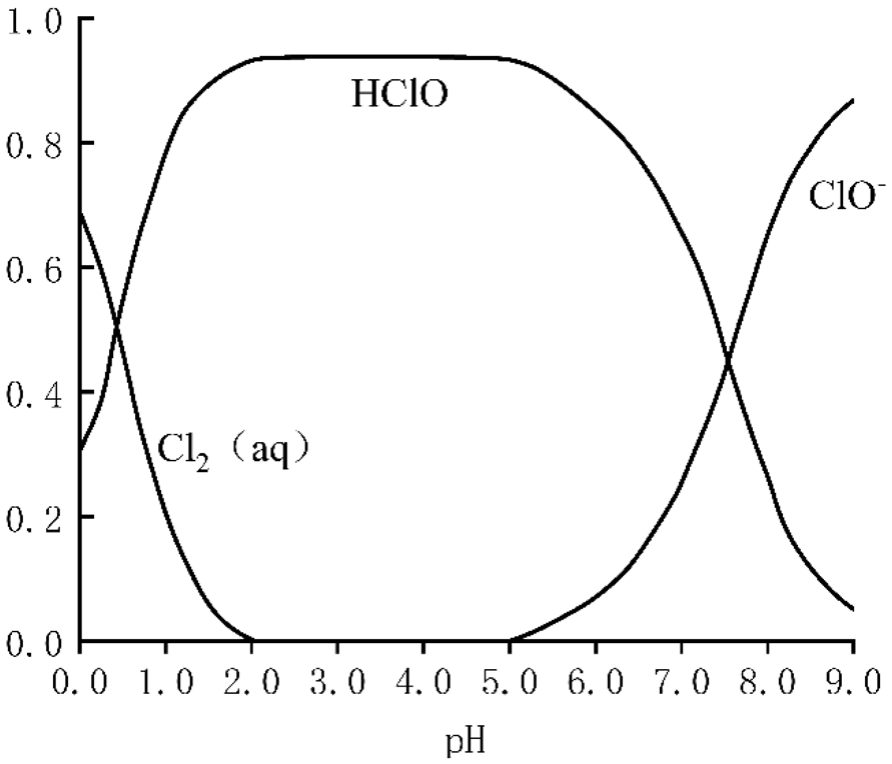
Solution pH and each component scores.

The HClO from electrolysis of NaCl has an oxidation potential (HClO^−^/Cl_2_≈1.61 V) and has the highest concentration in acidic solution. Under alkaline conditions, hypochlorous acid ions (ClO^−^) have the highest concentration but have a mild oxidation potential (ClO^−^/Cl^−^≈0.89 V). The relationship between the distribution of oxidizing substances in the electric field and pH is shown in Fig. 2, which means that the oxide components can be adjusted by manipulating the system pH. In a weakly oxidized environment, amino acids are deaminated in the presence of hypochlorite but do not cause degradation of the backbone^22^. In the process of electrolysis, to prevent excessive degradation and volatilization of electrolytes and keep the concentration of electrolytes stable, 0.1 mol/L NaOH was used to control the pH from 3 to 3.5 in the anodic region, and 0.1 mol/L HCl was used to control the pH from 9.0 to 9.5 in the cathodic region.

#### Determination of amino group content in the electrolyte

In a slightly acidic solution, ninhydrin is heated with amino acids to produce a blue-purple compound through oxidation and deamination^23^. The maximum absorption peak of this compound is at 570 nm wavelength, and the size of the absorption peak is proportional to the content of amino groups released by amino acids^24^. In the ninhydrin solution and ascorbic acid in buffer solution (pH = 5), the amino group content in the electrolyte can be determined by the standard curve of absorbance and GlyGl content.

Method for analysis of test solution: The electrolysis time lasted for 30 min and was repeated every 30 min for each experiment; after 3 h, a total of 8 parallel experiments had been performed. The electrolyte was poured out, and a small amount of deionized water was used to wash the electrolytic cell. The collected liquid was combined for constant volume analysis, and the quantity of amino groups was obtained according to a volume conversion.

#### Analysis of ^13^C NMR of electrolyte

^13^C NMR analysis of the GlyGl electrolyte after 90 min of electrolysis without a membrane was performed. D_2_O was used as the solvent, and a Bruker-E200 NMR spectrometer (Switzerland, Bruker) was used for comparison with the GlyGl solution.

### Electrolysis of gel

#### Electrolytic experiment

Anodic electrolysis under an anion membrane was performed. In the experiment, Gel was placed into the anodic area (Use Gel instead of GlyGl in Table 1). After Gel swelled, 100 mL of a 5 g/L solution was prepared at 65 °C and added to the anodic area with a supporting electrolyte NaCl concentration of 20 g/L. 100 mL of deionized water was added to the cathode, and the concentration of NaCl was 20 g/L.

Operation method: Electrolysis was carried out at 25 °C, 35 °C and 45 °C for 3 h, and the other conditions are shown in experiments 2.2.1 and 2.2.2.

#### Determination of the isoelectric point of the electrolyte

The isoelectric point is the characteristic parameter of amphoteric electrolytes, and the pI is changed by the change in the ratio of the Gel amino group and carboxyl group. Two experiments were carried out for Gel electrolysis at 1 h and 3 h. A Zeta Pals laser scatterer ZEN3600 (Malvern, UK) was used to test the zeta potential of electrolytic Gel solution samples at different pH values, and the isoelectric point pI was obtained by the curves.

#### Determination of the molecular weight

The molecular weight of Gel was determined after 1 h and 3 h of electrolysis by a gel permeation chromatography (GPC) system. The chromatographic conditions were as follows: TSK-GEL G-5000 PW xL column (7.8 mm × 300 mm) and G-3000 PW xL column (7.8 mm × 300 mm), 0.02 mol/L KH_2_PO_4_ as the mobile phase with a pH of 6.0, a flow rate of 0.6 mL/min, a column temperature of 35 °C, and a sample size of 20 μL.

#### Determination of the electrolyte viscosity

Because of the low concentration and small viscosity difference, the sample needs to be concentrated after electrolysis. In the process of electrolysis, two experiments were carried out for 1 h and 3 h. After removing the two anodic electrolytes and washing the cell with a small amount of deionized water, the electrolyte was collected in a 250 mL beaker. The beaker was placed in a vacuum oven and concentrated to 100 g at 50 °C (up to 5 g/L Gel). According to standard GB/T 12457-1990, the NaCl content in the polar electrolytes was determined by titration with silver nitrate, using potassium nitrate as an indicator. The pH of electrolytes determined was approximately 4.5. A 5 g/L nonelectrolytic glue solution was added to a solution with the same concentration of NaCl as the electrolyte, and the pH was adjusted with HCl to be the same as the electrolyte that was used as the contrast solution.

The relative viscosity of the liquid to be measured was measured by an Ubbelohde viscometer^25^. Its purpose was to measure the flow time T of the liquid in the capillary at a certain temperature to calculate the viscosity of the liquid by Poisson's formula:

1 $$\eta = \frac{{\pi r^{4} pt}}{8Vl}$$

where *r* is the capillary radius of the Ubbelohde viscometer, *V* is the volume of liquid flowing through the capillary, *l* is the effective length of the capillary, and *p* is the pressure difference between the two ends of the viscometer.

Viscosity determination: The time it took for the standard liquid and the sample liquid to flow through the capillary of the Usher viscometer was measured. Rearranging Eq. (1), the two equations are divided to obtain Eq. (2). Using the known viscosity value of the standard liquid, the viscosity of the liquid to be measured is calculated as follows:

2 $$\frac{{\eta_{1} }}{{\eta_{2} }} = \frac{{p_{1} t_{1} }}{{p_{2} t_{2} }}$$

Where *η*_1_ and *η*_2_ are the viscosities of the standard liquid and the liquid to be measured, respectively, *t*_1_ and *t*_2_ are the flow times of the standard liquid and the liquid to be measured in the capillary, respectively, and *p*_1_ and *p*_2_ are the pressure differences between the standard liquid and the liquid to be measured at both ends of the capillary. For the Ubbelohde viscometer, *p* = *ρ*gh, *ρ* is the liquid density, g is the acceleration due to gravity, and h is the distance between the liquid level and the capillary end. The liquid level decreases gradually until time t, so h should be the "average" distance between the liquid level and the capillary end at time t, and h is roughly the same for different liquids. Then, the above equation becomes Eq. (3).

3 $$\eta_{2} = \eta_{1} \frac{{\rho_{2} t_{2} }}{{\rho_{1} t_{1} }}$$

Distilled water was used as the reference liquid in the experiment, and the viscosity of distilled water at 25 °C (0.89 × 10^4^ mPa·s) was obtained but could be ignored. The temperature of the sample was adjusted to 25 °C, the pH value determined was approximately 4.5, and the sample was measured after placing for 10 min.

#### Determination of the transparency of the electrolyte

5 mL of the Gel electrolyte used to measure the viscosity in Experiment 2.3.3 was placed in a 50 mL volumetric flask and diluted with distilled water to the scale line. After shaking, a UV-2501 UV–Vis spectrometer (Shimadzu, Japan) was used to measure the absorbance of the sample, and the color and transparency were characterized at wavelengths of 500 nm and 620 nm^26^.

### Electrolysis of 3DC

#### Electrolytic experimental operation

Electrolysis with an anionic exchange membrane at the anode was adopted. 10 g 3DC was cut into ≤ 10 mm × 50 mm blocks and placed in the anodic area. Then, 200 mL deionized water was added, and the concentration of supporting electrolyte NaCl was 20 g/L. At the same time, 20 g/L Na_2_SO_4_ was added as a collagen swelling inhibitor.

Operation method: At 35 °C, four electrolytic experiments were carried out at 1.0 h, 2.0 h, 4.0 h and 6.0 h. After the electrolytic experiment was completed, the liquid and 3DC were refrigerated and reserved for later use.

#### Analysis of the electrolyte TOC and TN

In addition to protein or collagen fiber, 3DC contains a large amount of hyaluronic acid and a mixture of amino polysaccharides. Organic matter degradation and dissolution into the electrolyte were investigated by analyzing the contents of TOC and TN in the electrolyte. The electrolysis samples taken at 1.0 h, 2.0 h, 4.0 h and 6.0 h were centrifuged at 10 MPa for 10 min. After the supernatant was diluted, the total nitrogen content of the TN of the electrolytes was determined by an automatic Kjeldahl nitrogen determination instrument (K1100, Jinan Hineng Instrument Co., Ltd.). The TOC of the electrolyte was measured with a TOC analyzer (Liqui TOC II, Elementar, Germany) and compared with the blank. In the blank experiment, the liquid was agitated for 1.0 h, 2.0 h, 4.0 h and 6.0 h in the absence of an electric field. The TOC and TN were determined after the liquid was removed by centrifugation and separation with the same electrolytic liquid phase. Increases in the TOC and TN can be observed both in samples that underwent electrolysis and in those that did not undergo electrolysis.

#### Observation of electrolytic 3DC tissue

The 3DC sample before electrolysis and the 3DC samples at 2.0 h, 4.0 h and 6.0 h after electrolysis were freeze-dried, and the tissue changes of the collagen fibers were observed under a scanning electron microscope (SEM) (JSM-7500F, JEOL) after being sliced by a refrigerating slicer.

### The statement about experimental animals

The goat skin used in our manuscripts “Exploration of Collagen Cavitation Based on Peptide Electrolysis” comes from our research institute “The Key Lab. of Leather Chem. and Engin. of Ministry of Education, Sichuan University”, which is a research institute to study how to better use animal resources to serve human beings. Of course, all animal skins for experiment come from the places where animals are raised for food, and then were purchased from a slaughterhouse with national animal slaughter qualification and meet the ARRIVE guidelines. The goat skins in this experiment came from the Chengdu tannery, which is a slaughterhouse in Chengdu, China. We specifically attest to this.

## Results and discussion

### Analysis of the GlyGl electrolytic results

#### Electrolytic time and amino group content

**Figure 3:**
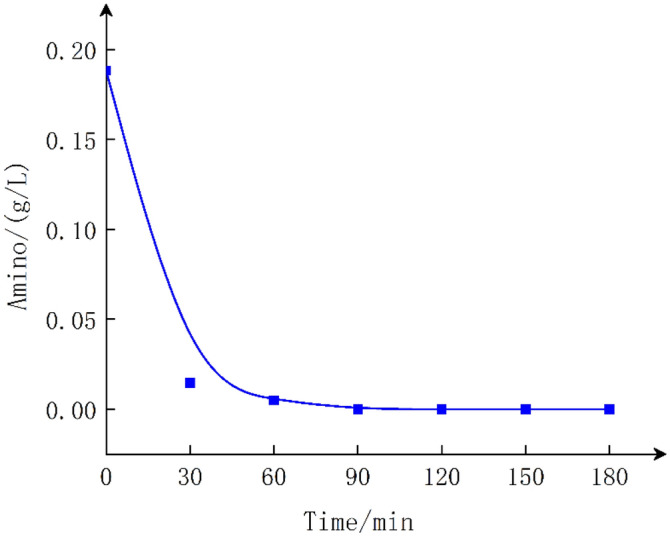
NH_3_-N content to electrolytic time without membrane.

Electrolysis of #1 (diaphragm-free GlyGl): Samples were taken every 30 min. Amino group determination is shown in Fig. 3. The initial 100 mL electrolyzed sample contains 5 g GlyGl, in which the amino acid content is 184 mg/L. Under a current of 15 mA·cm^−2^ for 1 h, the amino acid of GlyGl nearly completely disappeared under the electric field. Since the kinetic Kjeldahl nitrogen-determination apparatus cannot determine NO^2−^ and NO^3−^ ions, it can be determined that the amino group in GlyGl is completely oxidized in the absence of a membrane.

**Figure 4:**
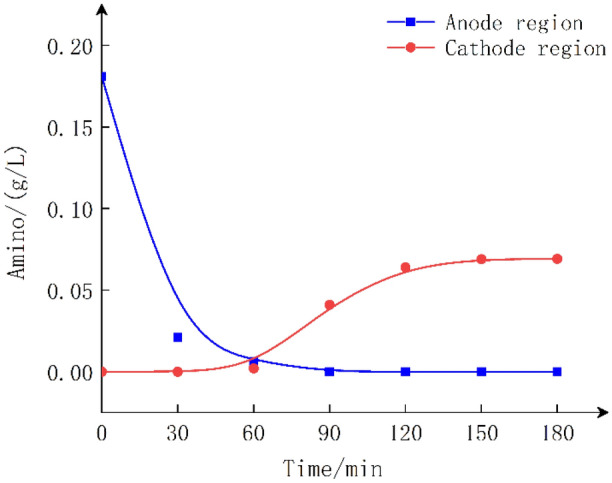
GlyGl migration *vs* time in the anode region under cationic membrane.

Electrolysis of #2 (GlyGl in the anode region of the cationic membrane): Fig. 4 shows that NH^4+^–R–COOH is formed when GlyGl is in the anode region and can reach the cathode region through the cationic membrane under an electric field. Conversely, when GlyGl reaches the cathode, GlyGl forms NH_2_–R–COO^−^, which cannot penetrate the cationic membrane. At 3 h, only 40% of the amino group of GlyGl had penetrated the cationic membrane.

**Figure 5:**
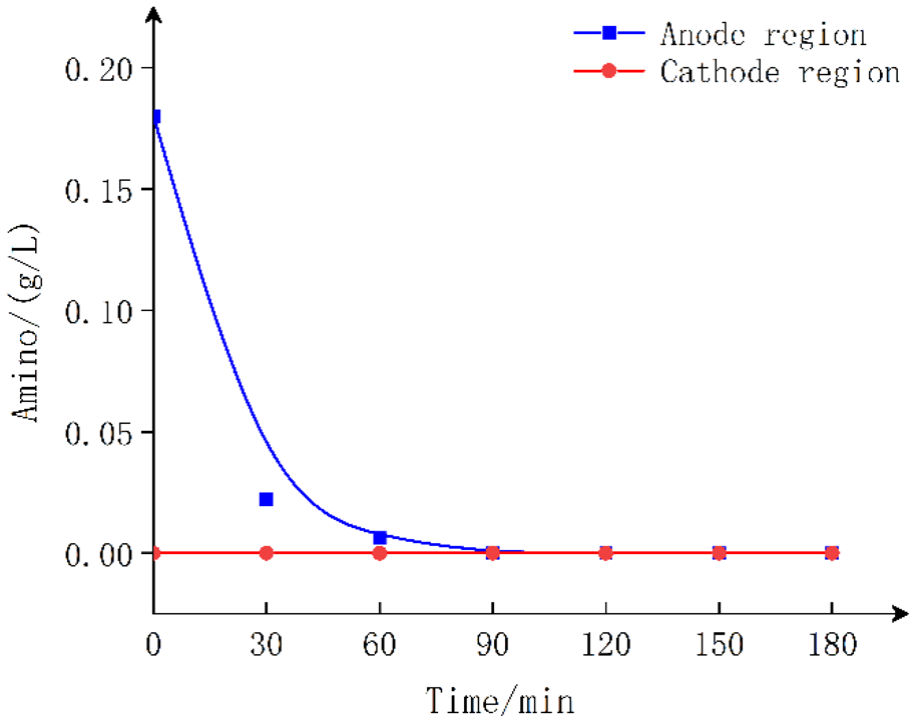
GlyGl migration *vs* time in anode region under anionic membrane.

Electrolysis of #3 (GlyGl in the anode region of the anion membrane): Fig. 5 shows that NH^4+^–R–COOH is formed when GlyGl is in the anode region, which cannot reach the cathode region through the anion membrane under an electric field, and the amino group in GlyGl is rapidly oxidized and converted at the anode region^27^. Contrast this with Fig. 3, the rapid disappearance of the amino group indicates that no GlyGl or amino group permeates the anion membrane during the electrolysis process.

#### ^***1****3*^*C NMR analysis of GlyGl electrolyte*

**Figure 6:**
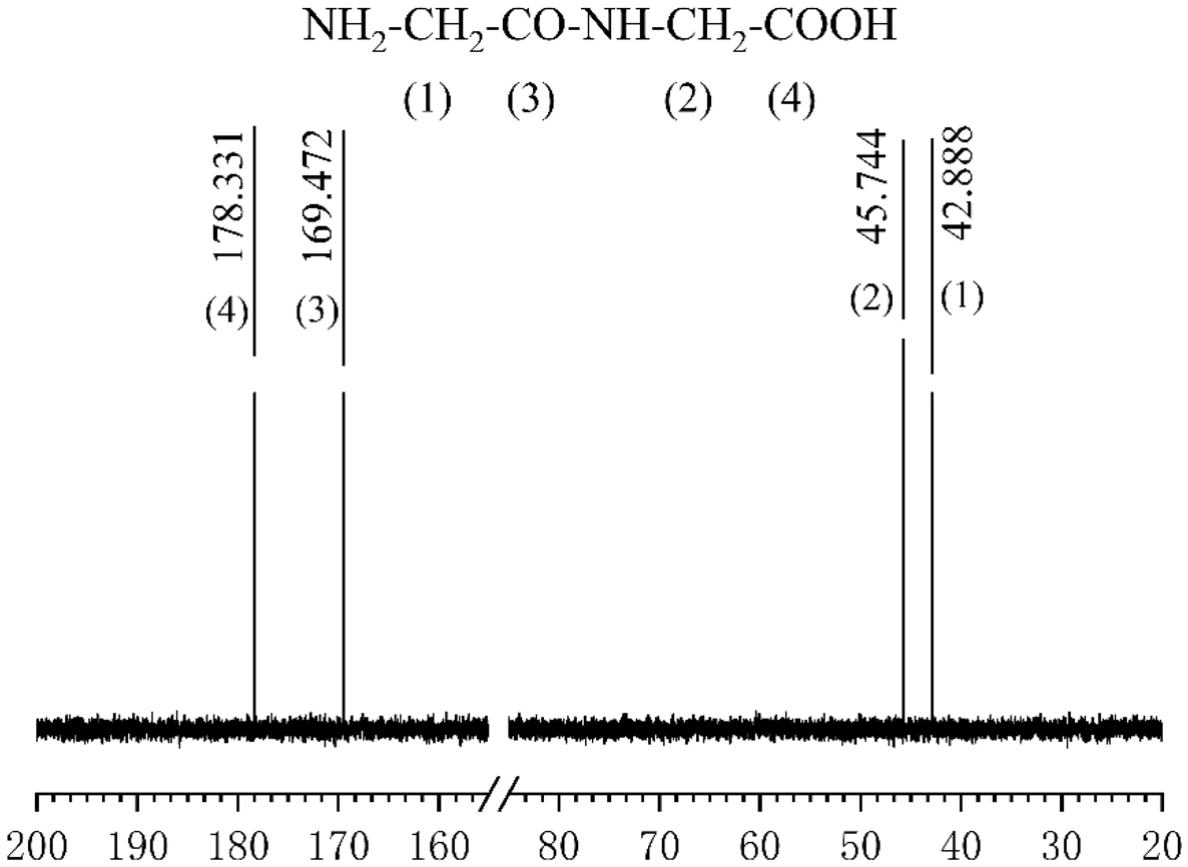
^13^C NMR spectrum of GlyGl before electrolysis.

**Figure 7:**
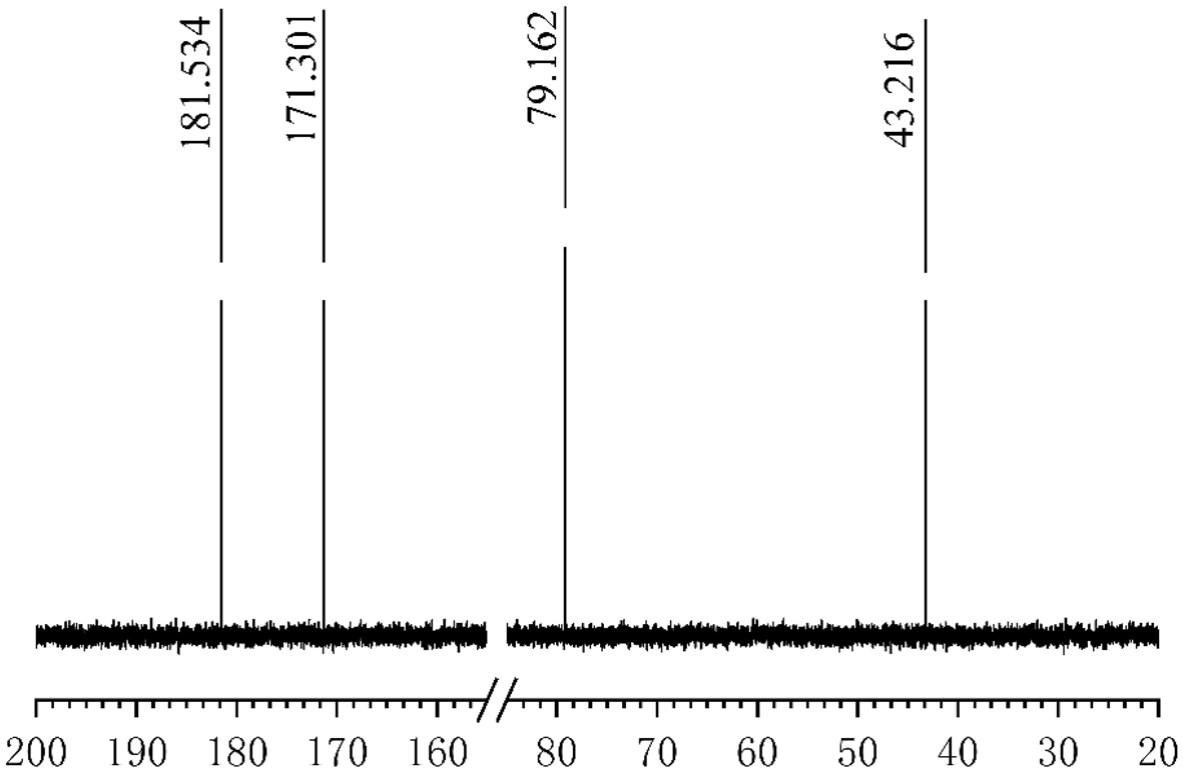
^13^C NMR spectrum of GlyGl after 3 h of electrolysis.

To determine the structure of the electrolyte, the electrolytic solution was sampled after the disappearance of the GlyGl amino group and analyzed using a BRUKER-E200 nuclear magnetic resonance spectrometer (BRUKER, Switzerland). The ^13^C NMR spectra of GlyGl in various electrolytic states were obtained. The results compared to the ^13^C NMR spectra of blank GlyGl are shown in Figs. 6 and 7.

According to the analysis results in Figs. 3, 4 and 7, after 90 min of electrolysis, the carbon connecting the amino group has largely been displaced, resulting in the disappearance of the amino group under the action of HCO. There is considerable kinetic evidence for the chlorine transfer reaction of chloramines^28^, which forms two kinds of ammonia chloride structure (Cl–NH–CH_2_–CON(Cl)–CH_2_COOH), which there was no breaking of peptide bonds^29^. The further oxidation and hydrolysis reaction of chloramine can break the peptide bonds and produce new carboxyl groups^30^.

### Analysis of the gel electrolytic results

#### Change of isoelectric point

The results of determining pI between the nonelectrolyzed Gel solution and gelatin solution electrolyzed for 1 h and 3 h are shown in Table 2. The concentration of the nonelectrolyzed Gel solution was 10 g/L.

**Table 2:** pI of the Gel electrolyte for the anode area with an anionic membrane.

	No electrolytic gelatin	Gel electrolysis time	
		1 h	3 h
pI	5.2	3.8	3.2

Table 2 shows that pI decreased with prolonged electrolysis time of the Gel solution. It can be shown that the amino group content decreases, and it can be concluded that the amino group content after electrolysis with Gel is obviously affected by HCO, and a special electrochemical degradation-modified Gel is formed^31^. The difference between the samples at 1 h and 3 h is caused by the consumption of NaCl and the reduction of oxidizable amino groups.

#### Change in molecular weight

In Table 3, Gel_E-1_ and Gel_E-3_ represent the gelatin samples electrolyzed for 1 h and 3 h, respectively. The results show that with prolonged electrolysis time from 1 to 3 h, the molecular weight of Gel rapidly decreased, the dispersibility markedly increased, and the macromolecular fragments decreased, which shows that electrolytic time is also a factor that cannot be ignored.

**Table 3:** GPC analysis of Gel molecular weight before and after electrolysis.

Sample	*M*_*w*_	*M*_*n*_	*d*_*p*_
Gel	5.20 × 10^4^	2.72 × 10^4^	1.901
Gel_E-1_	4.32 × 10^4^	2.19 × 10^4^	2.186
Gel_E-3_	1.23 × 10^4^	4.99 × 10^3^	3.459

#### Change in viscosity

Table 4 shows the viscosity changes between 1 and 3 h of electrolysis at 25 °C, 35 °C and 45 °C. Compared with the blank samples, the viscosity of the Gel solution after electrolysis for 1 h and 3 h varies greatly under various conditions and can be greatly reduced by electrolysis. Under the electric field, the molecular orientation of gelatin increases the accessibility of oxidation and the molecular destruction is rapid^32^.

**Table 4:** η of the Gel electrolyte solution (pH = 4.5).

Electrolysis time	(± 0.005)*η*/mPa·s		
	25 °C	35 °C	45 °C
0	2.685	2.101	1.505
1 h	1.329	1.059	0.952
3 h	0.797	0.650	0.579

In general, temperature has little effect on electrolysis. For the macromolecular Gel electrolyte, the molecular expansion can be improved, and the polar bond is weakened with increasing temperature, which can be illustrated by the change in *η* from 25 °C to 35 °C. However, the viscosity decreases slowly at 45 °C, indicating that 35 °C can be the ideal temperature for experimental electrolysis, especially for electrolysis of 3DC at higher temperatures, where the influencing factors of the electrode are increased.

#### Change in transmittance

The transmittance of the electrolytic Gel solution at 450 nm and 620 nm was measured at 35 °C, as shown in Table 5. Table 5 shows that the transparency of the Gel solution after electrolysis is lower than that of the original sample, and the transparency of the Gel electrolyte after 3 h is higher than that of the Gel electrolyte after 1 h. It can be shown that with the prolongation of the electrolysis time, the water solubility of the Gel will increase, which is consistent with the decrease in viscosity shown in Table 3. ^33^.

**Table 5:** Transparency of the Gel electrolyte.

Wavelength	No electrolysis	Electrolysis time	
		1 h	3 h
450 nm	97.7	93.5	97.6
620 nm	99.7	97.9	97.9

### Analysis of the electrolytic effect of 3DC

After treatment prior to tanning, organic noncollagenous substances were still present in the 3DCs obtained from animal skin. In the electrolysis method, using the small functional volume and high activity of electrons, collagen and noncollagen substances, such as hyaluronic acid, chondroitin sulfate, keratan sulfate, and a small amount of other substances, could be made. These materials can be dissolved by electrode gradation. In light of the disappearance of amino groups and the decrease in viscosity of the Gel solution in the above electrolytic experiment, it can be speculated that the amount of protein or peptide dissolved is difficult to determine by analyzing the amino groups, but it can be comprehensively characterized by TOC and TN.

#### TOC and TN in the electrolyte

The sample electrolytic solution was regularly removed for centrifugal filtration and diluted 50 times. Then, the TOC and TN in the waste liquid were determined. Table 6 shows that there is a large increase in TOC and TN in the electrolyte under an electric field, which indicates that the dissolution of organic matter is noticeable. However, the dissolution rate of organic matter reached a maximum within 1 ~ 3 h and then slowed down. This change in rate is associated with the decrease in NaCl over time, although 20 g/L sodium sulfate in an electric field can also produce sulfate radicals (·SO_4_^−^) with functional oxidizing particles^34^. Both the TOC and the TN of blank 3DC samples increased in the absence of an electric field, and the increase was very different from that after an electric field was applied.

**Table 6:** Total N and total C content of electrolytic solution and time.

Samples	Condition	1.0 h	2.0 h	4.0 h	6.0 h
TOC/(mg/L)	Electrolysis	757.1	1123.6	1216.1	1251.6
	No electrolysis	357.1	450.3	491.3	571.6
TN/(mg/L)	Electrolysis	164.7	263.5	307.3	320.5
	No electrolysis	30.2	33.2	35.2	36.6

The average nitrogen content of animal protein is 16%, and the carbon content is 50 ~ 60%; that is, the N/C weight ratio is 0.26 ~ 0.32. The N/C ratios in the 3DC electrolyte are 0.217 (after 1.5 h of electrolysis), 0.234 (after 3.0 h of electrolysis), 0.253 (after 4.5 h of electrolysis) and 0.256 (after 6.0 h of electrolysis). The results showed that protein dissolution increased with increasing electrolysis time. In the absence of an electric field, the N/C ranged from 0.084 to 0.064 in 1–6 h, indicating that the dissolution of polysaccharides slightly increased.

#### SEM comparison of electrolytic 3DC

**Figure 8:**
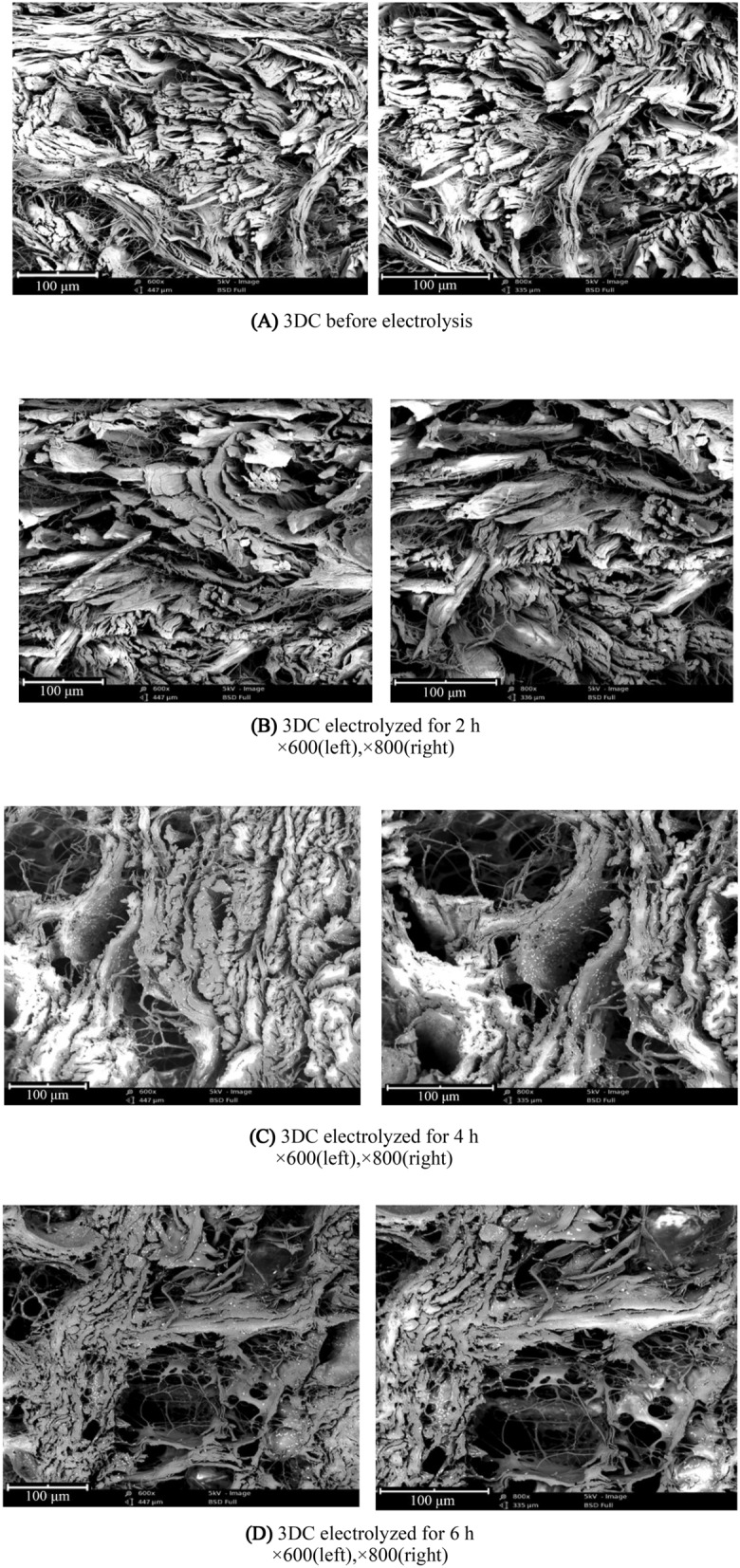
The SEM images of 3CD before electrolysis and electrolyzed for 2 h, 4 h, and 6 h.

The raw skin and skin blocks subjected to electrolysis for 2 h, 4 h and 6 h were freeze-dried, and the collagen fiber dispersion was observed under a scanning electron microscope after being sliced by a refrigerated slicer. The comparison of the SEM images is shown in Fig. 8 below.

The SEM images of the 3DC organizational structure at 2 h, 4 h and 6 h before and after electrolysis were compared. After electrolysis, the distance between collagen fiber bundles in 3DCs was larger, and the fibers were looser. After 2 h of electrolysis, the filamentous fibers between the pores of the fiber bundles almost disappeared, and the fiber bundles shrank. Images at 4 h and 6 h showed an increase in filamentous fibers. Two different 3DC structures can be obtained from electrolysis for 4 h and 6 h. It had an obvious cavitation effect.

## Conclusions

Using NaCl as the supporting electrolyte and oxidant, it was shown that the amino and peptide bonds of GlyGl were obviously modified in the anode region isolated by anion exchange membranes. The molecular weight of Gel could be degraded and the solution characteristics of Gel could be modified, which with the prolongation of electrolysis time, the viscosity of the solution decreases and the transparency increases under the action of an electric field within the anode region isolated by anion exchange membranes. The dissolution effect of organic matter in animal skin (3DC) was characterized by TOC and TN analysis of the effluent, and the organization structure of 3DC was observed after electrolysis. The results show that the electrolysis of 3DC can reduce the density, which is a special cavitation effect. Electrochemical treatment of animal skin collagen is a new method to prepare porous animal skin biomaterials.
